# Synthesis and characterization of DGEBA composites reinforced with Cu/Ag modified carbon nanotubes

**DOI:** 10.1016/j.heliyon.2019.e01733

**Published:** 2019-05-29

**Authors:** Anila Iqbal, Aamer Saeed, Ayesha Kausar, Muhammad Arshad, Jamaluddin Mahar

**Affiliations:** a Department of Chemistry, Quaid-i-Azam University, Islamabad, 45320, Pakistan; b National Centre for Physics, QAU Campus, Shahdra Valley Road.P.O. Box No. 2141, Islamabad, 44000, Pakistan

**Keywords:** Analytical Chemistry, Materials chemistry

## Abstract

Carbon nanotubes (CNTs) are among the strongest and stiffest contender to be used as filler to elevate the properties of epoxy. The aim of this research work is to evaluate the structural, thermal, and morphological properties of multiwalled carbon nanotubes (MWCNTs) hybridized with silver, copper and silver/copper nanoparticles (Ag/CuNP) obtained via chemical reduction of aqueous salts assisted with sodium dodecyl sulphate (SDS) as stabilizing agent. The MWCNTs/NP was further incorporated in DGEBA (epoxy) using ethyl cellulose as hardener. Scanning electron microscopy (SEM) reveals micro structural analysis of the MWCNTs/NP hybrids. The Fourier transform infrared (FTIR) spectra prove the interactions between the NP and MWCNTs. Thermogravimetric analysis (TGA) shows that the MWCNTs/NP hybrids decompose at a much faster rate and the weight loss decreased considerably due to the presence of NP. X-ray diffraction (XRD) confirms the formation of NP on the surface of MWCNTs and X-ray photoelectron spectroscopy (XPS) confirms the full covering of MWCNTs/NP hybrids with DGEBA.

## 1 Introduction

One of the great interests in the field of advanced composites is the incorporation of nanomaterials in the polymer matrix. The ideal reinforcement with outstanding properties for polymers such as epoxy resin is multiwalled carbon nanotubes (MWCNTs) although a lot of other reinforcements are also investigated such as nanodiamonds (Zhang et al., 2017), Carbon fiber (Kim et al., 2017) and graphene oxide (Zhang and Cho, 2018). These composites formed by the incorporation of MWCNTs possess good mechanical properties, excellent electrical and high thermal conductivities then the bulk materials (Fu et al., 2010; Chakraborty and Roy, 2012; Vajtai, 2013). Type of Polymer matrix, MWCNTs and the interphase region between the matrix and the MWCNTs are the important contributing factors to the properties of the composites (Cui et al., 2003; Fiedler et al., 2006; Moniruzzaman and Winey, 2006; Li et al., 2009; Sahoo et al., 2010). Normally MWCNTs have high tendency to aggregate, which is one of the main hindrance in the uniform dispersion of MWCNTs in the polymer matrix and may affect the properties of the composites (Baig et al., 2018) Functionalization of MWCNTs helps in the dispersion (Gojny and Schulte, 2004) but the structure of the MWCNTs is compromised due to harsh treatments. The type of interactions which are supposed to exist between the polymer and MWCNTs are covalent and non-covalent or physical interactions. MWCNTs are one of the main allotropes of carbon with sp2 hybridized carbon atom forming the main structure (Iijima, 1991). MWCNTs are graphene layers, rolled up cylindrically with diameter in nanometer range. The presence of sp2 carbon-carbon bonds gave special structural, mechanical, electronic, and optical properties to MWCNTs (Treacy et al., 1996; Chen et al., 1999; Ma et al., 2008). High level techniques are used nowadays to study the magnetic and electronic structure of carbon nanotubes using X-ray absorption and magnetic dichroism spectroscopy (Gautama et al., 2011) and to detect ferromagnetism in CNTs (Friedman et al., 2010).

The use of CNTs at industrial level in comparison to the conventional materials is still not possible due to difficulties related to the production, dispersion, purification for industrial applications. The other problems which are of high concern is the less knowledge of mechanism of CNTs synthesis, commercialization of CNTs and the environmental and personal safety. Along with all these issues the CNT industry is growing quickly, and different kinds of CNTs based on required parameters are easily available (Zhang et al., 2013). A lot of scientific research also includes the theoretical and experimental study of the interface of such types of materials which is beneficial to enhance the range of application (Khoddam et al., 2018).

Another emerging class of nanomaterials is the metal nanoparticles (NP), having unique properties and remarkable difference then the bulk material. Among metal NP some of the noticeable contenders are palladium (Pd), gold (Au), copper (Cu), platinum (Pt), and silver (Ag) NP (Larrude and Costa, 2014). In recent years, detailed studies have been carried out to integrate the properties of MWCNT and NP and there potential was explored in different fields. This results in the creation of a new class of hybrid materials, which exhibit combined characteristics of both the nanomaterials with unique and much better properties. The high aspect ratio of MWCNTs which is due to small diameter and longer length makes it a good candidate to be use as templates for the assemblies of NP. The functionalization of MWCNTs with NP not only improves their properties but also elevates its applications (Ma et al., 2008). Silver nanoparticles (AgNP) are one of the best contenders due to its excellent properties and wide range of application such as in biosensors (Yu et al., 2012), optical limiters (Chin et al., 2005), antimicrobial agents (Yuan et al., 2008), catalysts (Wang, ZHU et al., 2008), metal adsorbents (Ramana et al., 2013) and advanced composites (Xin and Li, 2011). Copper NP (CuNP) is another very important and potential candidate for the functionalization of MWCNTs not only due to its wide range of applications but also due to much better and excellent properties. CuNP have been used for waste water treatment as disinfectant (Ruparelia et al., 2008) and show effective antibacterial activity, when stabilized on carbon, polymers, sepiolite, and polyurethane foam. Due to the large surface to volume ration of CuNP, their potential as catalyst for dye degradation has also been exploited. Fluorescence quenching, dye deaggregation, dye aggregation, and fluorescence enhancement may also be caused by CuNP along with its use in biosensing and biolabelling (Mandal and De, 2015). CuNP based nanomaterials have been use for its various conducting applications (Hokita et al., 2015).

There are various wet and dry techniques available for the synthesis of MWCNTs/NP hybrids. Dry chemical methods involve the physical vaporization of solid precursor materials, producing charged NP which are directly assembled on the surface of MWCNTs. Vapor deposition (Chin et al., 2005), gamma irradiation (Reddy et al., 2006), thermal decomposition (LeeáTan, 2001) and electrostatic force directed assembly (ESFDA) (Li et al., 2006) are few of the dry methods. However, commonly wet methods are used to deposit NP on the surface of MWCNTs as they are relatively inexpensive and fast. In addition, control over morphology, size distribution and the dimension of NP are yet another advantages of wet chemistry methods (Ghalkhani et al., 2009). In wet chemistry methods, metal salt precursors undergo chemical reduction in the presence of MWCNTs and the NP formed are directly deposited on the surface of MWCNTs (Liu et al., 2006; Sepahvand et al., 2008; Alimohammadi et al., 2012). Alternatively, the NP can be prepared as metal colloids and then deposited onto the MWCNT surface through covalent bonding by using organic fragments (Lee et al., 2011). Strong covalent bonds or weak intermolecular bonds such as pi-pi stacking, Vander Waal interactions, hydrogen bonding, hydrophobic interactions or electrostatic interactions are responsible for the interactions between the NP and the MWCNTs surface (Georgakilas et al., 2007). These weak interaction between the NP and MWCNTs surface as well as agglomeration of the NP hinders the deposition of NP on the MWCNTs surface (Ma et al., 2008; Xin and Li, 2011; Ahmadpoor et al., 2013). In present work, a simple reduction method is used to synthesize MWCNTs/Ag, MWCNTs/Cu and MWCNTs/Ag/Cu hybrids. During synthesis *N,N*-dimethylformamide (DMF) is used as reducing agent and sodium dodecyl sulphate (SDS) acts as surfactant. These synthesized hybrids are then incorporated in DGEBA using ethyl cellulose as hardener i.e. MWCNTs/Ag/DGEBA, MWCNTs/Cu/DGEBA and MWCNTs/Ag/Cu/DGEBA. Following this, the structural, morphological and thermal properties of MWCNTs/NP hybrids and MWCNTs/NP/DGEBA hybrids were examined using various characterization techniques.

## 2 Experimental

### 2.1 Materials

Diglycidyl ether bisphenol A (DGEBA), sodium dodecyl sulfate (SDS), silver nitrate (AgNO_3_), copper nitrate (Cu(NO_3_)_2_ .3H_2_O), ethyl cellulose, dimethylformamide (DMF) tetrahydrofuran (THF) and hydrochloric acid (HCl) were procured from Fluka. Nitric acid (HNO_3_) was obtained from Aldrich and was used as received.

### 2.2 Measurements

Infrared (IR) spectra were recorded using Fourier transform infrared (FTIR) Spectrometer, Model No. FTSW 300 MX, manufactured by BIO-RAD (California, USA) with 4 cm^−1^ resolution. Field emission scanning electronmicroscopy (FESEM) of freeze fractured samples was performed using JSM5910, JEOL Japan. Thermal stability was verified by METTLER TOLEDO TGA/SDTA 851 thermogravimetric analyzer using 1–5 mg of the sample in Al_2_O_3_ crucible at a heating rate of 10 °C/min. X-ray diffraction (XRD) technique was carried out by using Bruker, D8 advanced at the scan range of 5°–80°. The XRD was operated with CuK_α_ radiation source (λ = 1.54056Å), generated at 40 kV and 40mA. X-ray photoelectron spectroscopy (XPS) measurements have been performed using a Scientia-Omicron system equipped with a micro-focused monochromatic Al K-Alpha (1486.7 eV) X-ray source having spot size of 700-micron. The source was operated at 15 keV with constant analyzer energy (CAE) 100 eV for survey scans and 20 eV for high resolution scans. The Charge neutralization was applied using a combined low energy/ion flood source to avoid the charging effects. The data acquisition was done with Matrix software and analysis was performed with Igor pro along with XPS fit procedures. The Curve fitting of high-resolution spectra was made utilizing the Gaussian-Lorentzian line shape after performing the shrilly background corrections.

### 2.3 Synthesis of MWCNTs

MWCNTs were prepared by the patented technology (Patent number: US2009208403).

### 2.4 Purification of MWCNTs

Raw MWCNTs were initially annealed at 400 °C for 0.5 h to remove any amorphous carbon content. In order to eradicate metal catalyst, which was incorporated inside the nanotubes during synthesis, MWCNTs were refluxed in HCl for 3h at ambient temperature.

### 2.5 Synthesis of MWCNT/NP hybrid

Initially the synthesized MWCNTs were functionalized with the transition metal nanoparticles.1g of purified MWCNTs was added to 200 mL of DMF and 0.4 g of SDS. The reaction mixture was stirred for 5 hrs, pH was maintained at ∼6 by 0.01M HNO_3_. 60 mL (0.1M) of AgNO_3_ was added drop wise to the final mixture and the temperature was maintained from 60-62 °C for 1 h, followed by 48 hrs stirring at room temperature. The product was filtered and washed with acetone. The same process was repeated for the formation of MWCNT/Cu hybrids using 60 mL (0.1M) of Cu(NO_3_)_2_ .3H_2_O. For the preparation of MWCNT/Ag/Cu hybrid 60 mL (0.1M) of AgNO_3_ and 60 mL (0.1M) of Cu(NO_3_)_2_ .3H_2_O were added in the above mentioned protocol.

### 2.6 Synthesis of DGEBA/MWCNTs/NP hybrid

For the synthesis of DGEBA/MWCNTs/NP Hybrid, 0.1 g of MWCNTs/Ag was added to 20 mL of THF and sonicate for 3hrs. Then 3g of DGEBA was added to 30 mL of THF and followed by sonication for 12 hrs. In next step the sonicated MWCNTs/Ag solution was added to the DGEBA solution and the mixture was further sonicated for 3 hrs. In 20 mL of THF, 3 g of ethyl cellulose was mixed and sonicated for 3 hrs. This ethyl cellulose solution was also added to the final reaction mixture and the final product was further sonicated for 3 hrs. The reaction mixture was poured in Teflon petri dish and was heated at 90 °C for 1 h and further heated at 50 °C for 48 hrs. The above mentioned method was also used for the synthesis of DGEBA/MWCNTs/Cu Hybrid and DGEBA/MWCNTs/Ag/Cu Hybrid using MWCNT/Cu and MWCNT/Ag/Cu complex respectively.

## 3 Results and discussion

### 3.1 Preparation of DGEBA/MWCNTs/Cu/Ag hybrids

**Fig. 1:**
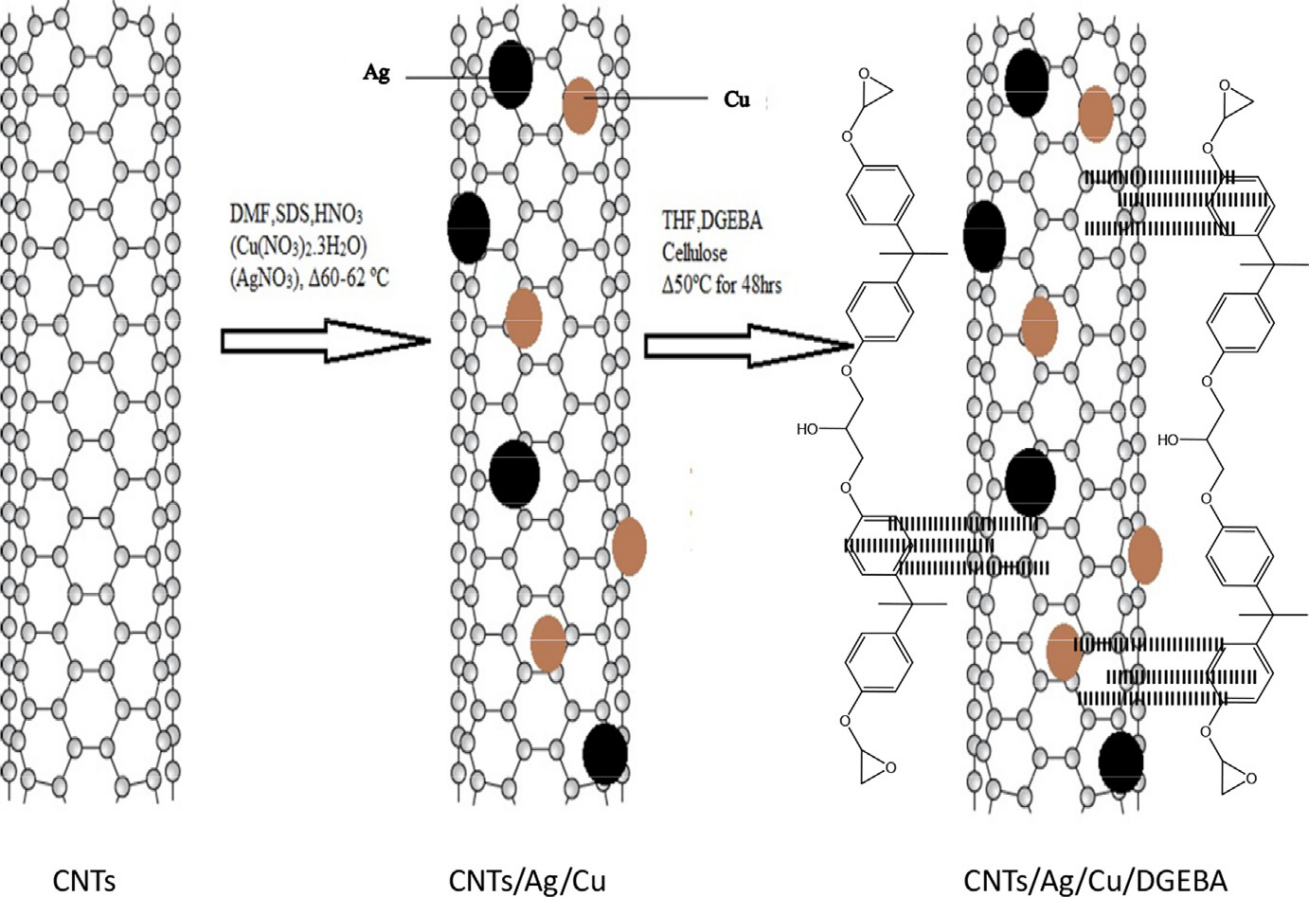
a: Synthesis of MWCNTs/Ag/Cu/DGEBA Hybrid.

**Fig. 2:**
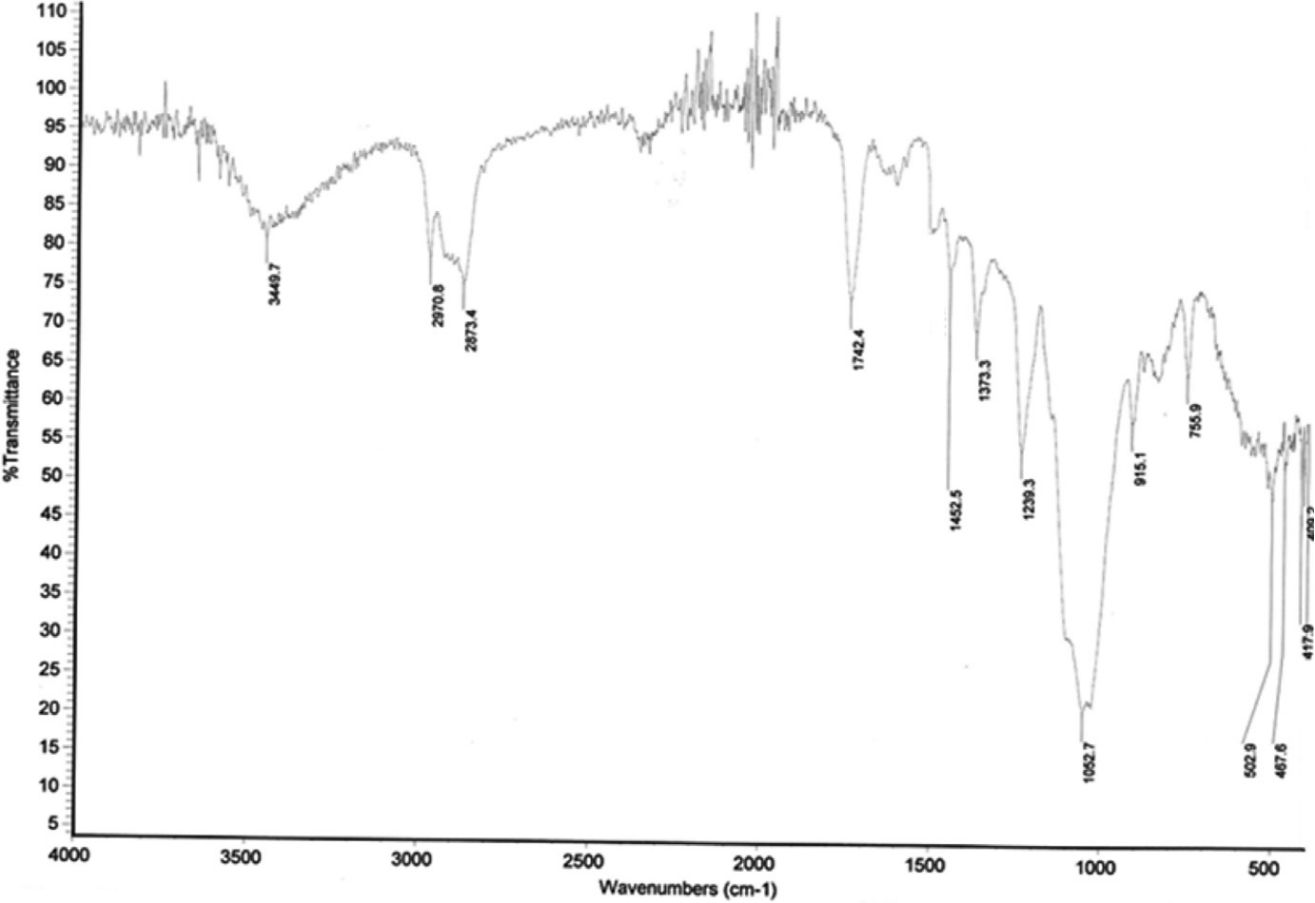
FTIR spectrum of MWCNTs/Ag/Cu/DGEBA hybrid.

Nanocomposites can be fabricated using different nanomaterials among which carbon nanotubes is a vital contender. To enhance the thermal properties of the nanocomposites, fabrication of nanocomposite must be carried out in a way that the other physical properties of the CNTs are not deteriorated. Silver nanoparticles (AgNP) and copper nanoparticles (CuNP) are good contender to enhance the thermal stability of the composite. The reason for synthesis of CNT/Cu/Ag is to determine the change in the properties of the prepared samples when both the conducting NP are added to the surface of the CNT. The thermal properties of all these samples were studied for comparison reasons. MWCNTs were purified in the first stage followed by the in-situ attachment of metal NP. AgNP and CuNP are formed on the surface of CNTs by the reduction of their salts. In the final stage the prepared samples were incorporated in DGEBA using ethyl cellulose as hardener. Synthesis is shown step by step in Scheme 1 (Fig. 1). The FTIR of CNTs/Ag/Cu/DGEBA is shown in Fig. 2.

Its shows the characteristic peak at 3449 cm^−1^ for O˗H stretching vibrations present in the epoxy. The vibrations at 2970 and 2873 cm^−1^ are representing C–H stretching and peaks at 1452 and 1373 cm^−1^ are due to C–H bending vibrations. The stretching vibrations for C=O which is present due to the oxidation of MWCNTs during purification was observed at 1742 cm^−1^. The presence of C–O bond is confirmed by the peaks at 1239 and 1052 cm^−1^. All these peaks confirm the presence of DGEBA and ethyl cellulose which was used as hardener during the synthesis. DGEBA is an epoxy resin, having epoxy group and is capable of converting into thermoset form. The epoxide groups of DGEBA join together by homopolymerization during the process of curing and results in the coupling as well as cross linking. The cleavage of the much strained epoxide ring results in the formation of covalent bond and as a result O–H bond is fashioned which is confirmed by peak in FTIR. The type of interactions between the DEGEBA and MWCNTs are physical and no chemical or hydrogen bonding are possible as the MWCNTs used in the synthesis are not functionalized. The types of physical interactions which are responsible for the attachment of NP and DGEBA on the surface of MWCNTs are Vander Waals forces, and weak intermolecular interactions such as pi-pi stacking and/or electrostatic attractions (Georgakilas et al., 2007). The tri-coordinated carbon in MWCNTs is arranged in non-planar orientation to form a cylinder. Each carbon atom has a mono-electronic p-orbital, which form an electronic cloud on the surface of the MWCNTs. The pi electrons in the aromatic frame work of DGEBA and those on the MWCNTs interact with one another through p-orbitals. This type of interaction is termed as pi-pi interaction or pi-pi stacking. The subsystems interacting through this type of stacking usually maintain their individuality (Tournus et al., 2005; Grimme, 2008).

### 3.2 XRD

**Fig. 3:**
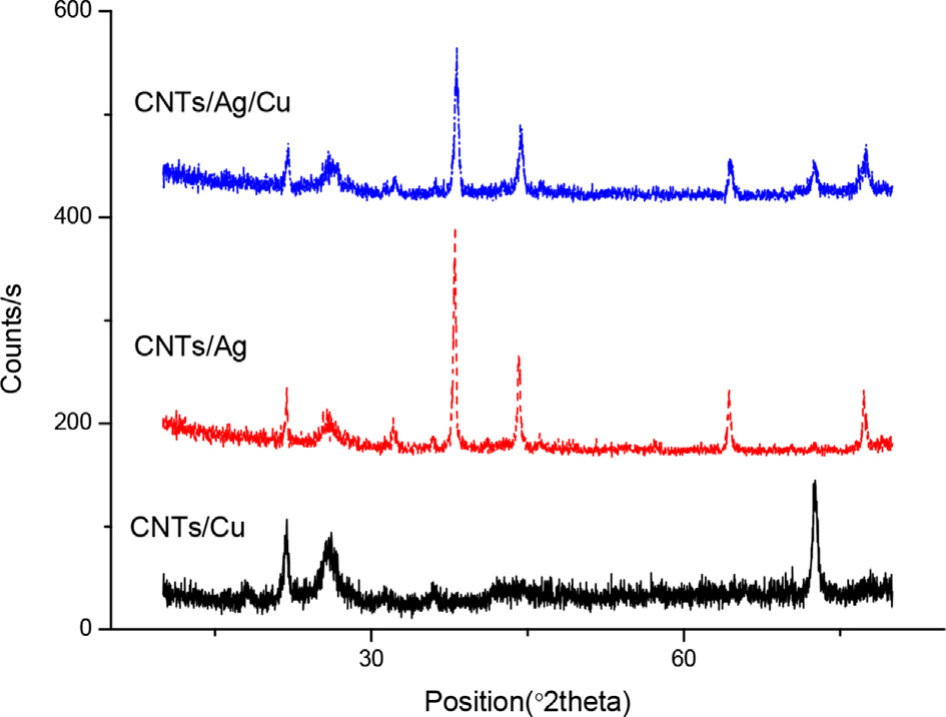
XRD of MWCNTs/NP complex.

The synthesized samples in the solid form were characterized by XRD and the results are shown in Fig. 3.

The XRD profile of DGEBA/CNTs/Cu, DGEBA/CNTs/Ag and DGEBA/CNTs/Ag/Cu shows the amorphous nature of the material, while CNTs/Cu, CNTs/Ag and CNTs/Ag/Cu composite exhibits characteristic peaks for the attached metal nanoparticles. In CNTs/Ag at diffraction angles (2θ) 38.00°, 44.14°, 64.35°, and 77.27° corresponding to (111), (200), (220), and (311) planes, respectively, and support the face-centered cubic (fcc) structure of AgNPs (Castle et al., 2011; Neelgund and Oki, 2011; Zhai, 2013). The crystalline fcc structure of AgNPs can be assigned to standard JCPDS Card 89–3722. Another intense peak corresponding to the MWCNTs was observed at 2θ is 25.92° (Zhai, 2013; Alshehri et al., 2016). The lattice plane reflection at all the peaks of Ag were used to calculate the average size of AgNPs according to the Debye–Scherrer equation, and the crystal size of AgNPs was estimated to be 345 nm. The peaks at 21.90°, 32.09°, 35.92° and 46.8° were due to impurities in CNTs. In case of CNTs/Cu peaks at 38.00°(111), 44.14°(111), 50.00°(200), and 73.22°(220) corresponds to the Cu nanoparticles present on the surface of CNTs. The average crystal size calculated was 155.92. CNTs/Ag/Cu represents all the peaks corresponding to Cu and Ag nanoparticles.

### 3.3 Morphology

**Fig. 4:**
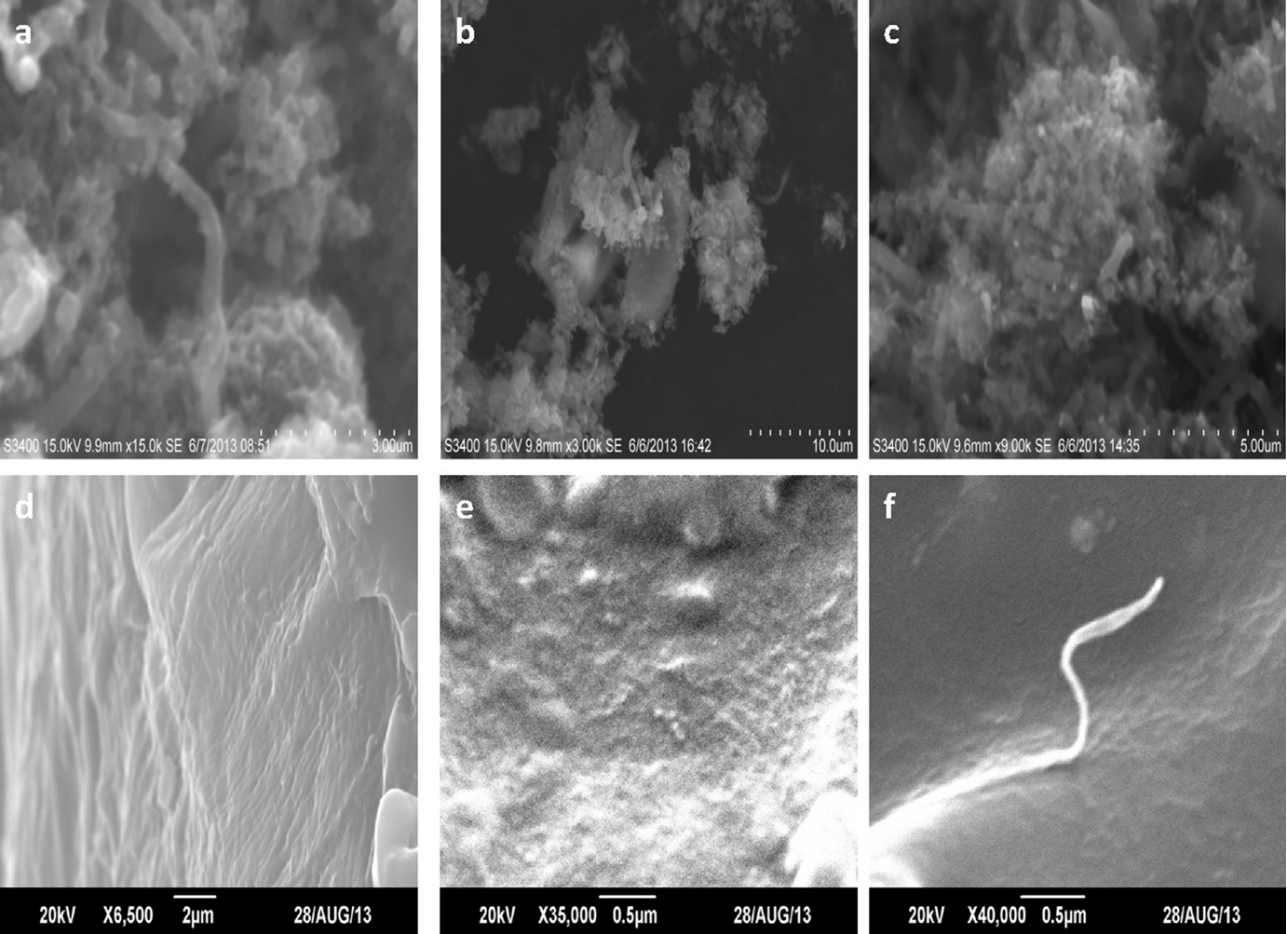
FESEM images of (a) MWCNTs/Ag(b) MWCNTs/Cu (c) MWCNTs/Ag/Cu (d) DGEBA/MWCNTs/Ag (e) DGEBA/MWCNTs/Cu (f) DGEBA/MWCNTs/Ag/Cu.

The morphology of the hybrids was determined by the Scanning electron microscopy (SEM) and results are represented in Figs. 4a, 4b and 4c.

The micrographs in Fig. 4(a) clearly showed the MWCNTs with attached bright and spherical AgNP. Similar type of CuNP can be seen in Fig. 4(b) where as both AgNP and CuNP are present on the surface of CNTs as shown in Fig. 4(c).

The SEM micrographs also showed that the CNTs are much bent and covered with high concentration of NP on the surface which may be due to the presence of defects on the surface of the graphitic network, created during purification. The steric effect is responsible for the formation of these aggregates of NP which later on converts into big clusters (Song et al., 2009). The NP are mostly accumulated on the surface of the MWCNTs because of the insufficient de bundling power of the anionic surfactant, and its Vander Waal attractions. The amount of SDS to act as a stabilizer is critical for the formation and accumulation of NP. In the case of MWCNTs/Ag/Cu Fig. 3(c) greater amount of aggregation which is due to insufficient amount of SDS used during experiment. Minimum amount of SDS is used in this study because the amount of surfactant influences the properties of the composites. Due to homogeneous nucleation, some of the NP are isolated which later can be observed to grow to larger aggregated NP as shown previously by Georgakilas et al. (Georgakilas et al., 2007). The attachment of NP on the surface of MWCNTs is attained by using SDS molecules by supramolecular self-assemblage mechanism. This type of assemblage is promoted by electrostatic interactions between the side walls of the MWCNTs and hydrophobic linear chains of SDS molecules (Neelgund and Oki, 2011). Fig 4(d-e) represents the SEM of DGEBA/MWCNTs/Ag, DGEBA/MWCNTs/Cu, DGEBA/MWCNTs/Ag/Cu respectively. It can be seen that it’s difficult to observe the MWCNTs on the surface of the material as epoxy cover all the surface of the composites, which is due to its high density. It can be deduced that all the MWCNTs/NP are covered fully by the epoxy.

The chemical method is used to synthesis the hybrids. The ratio of attachment of Cu and Ag nanoparticles on the surface of the CNTs is totally dependent on the concentration of the surfactant which is SDS. If the amount of SDS is increased the structure of MWCNTs of highly effected and defects are formed on the surface and if the amount of SDS is decreased to low level the attachment of NP on the surface is affected. So the reaction was optimized to the current SDS concentration to get the best results. The ratio of NP modified CNTs to DGEBA was optimized through a series of reaction by varying the amount of CNTs to DGEBA. The reported ratio represents the best results along with optimum thickness and flexibility of the hybrid film. As far as the TEM analysis is concerned its main purpose is to provide high magnification images of the internal structure the samples. The results provide information about the crystalline structure, contamination etc. The current research work is mainly focused on the synthesis of DGEBA nanocomposites and to study the dispersion of CNTs within the DGEBA matrix. The SEM micrographs clearly show that the CNTs are fully dispersed and no stranded or bundled CNTs are found as the SEM of the cracked surface are discussed Fig 4(d-e) so SEM provide sufficient evidence.

**Fig. 5:**
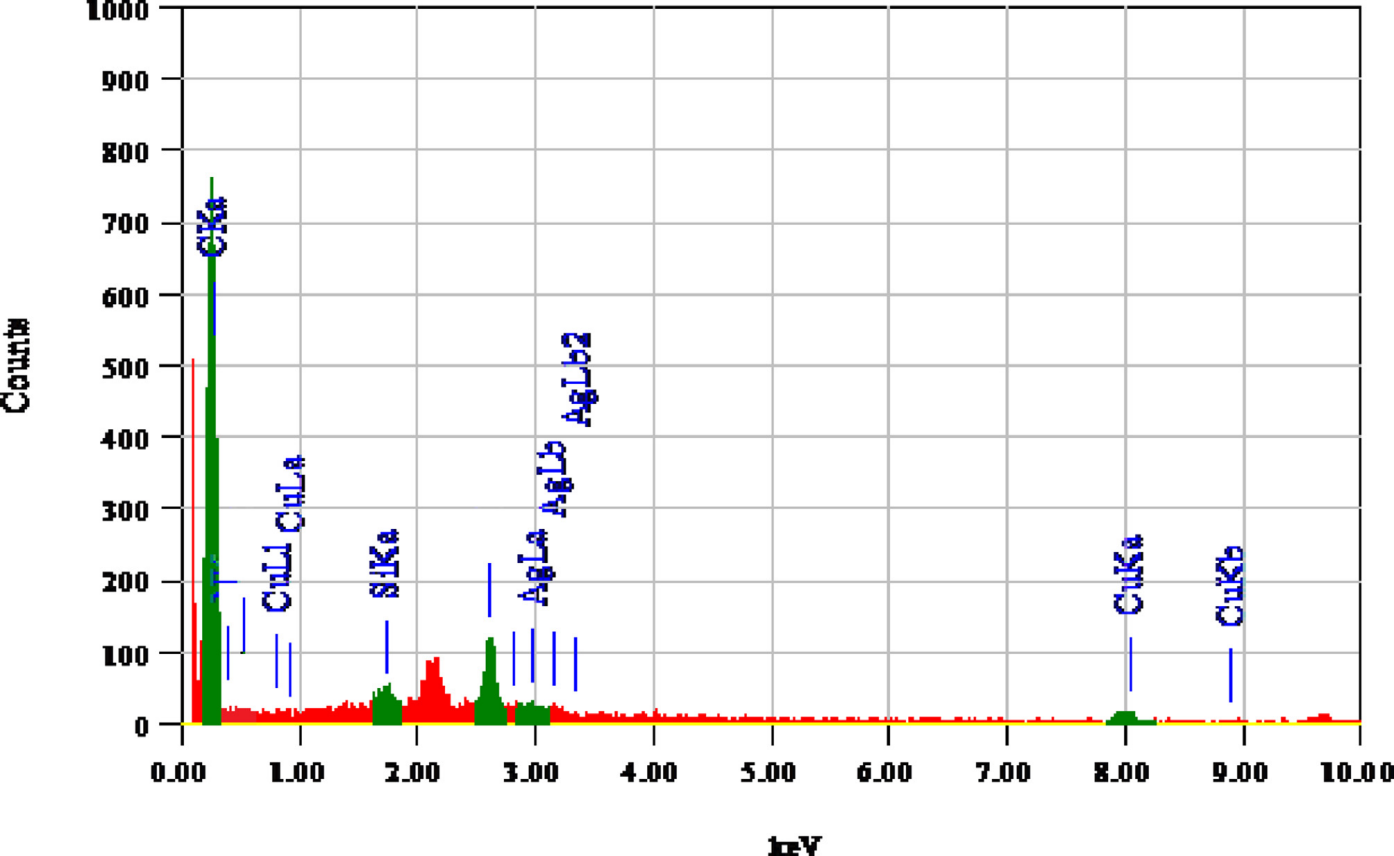
EDX of MWCNTs/Ag/Cu hybrid.

EDX spectrum is shown in Fig. 5 which represents an intense signal of carbon which is due to the CNTs present in the samples. The signals for AgNP and CuNP are also shown in the spectrum [42].

### 3.4 Thermal analysis

**Fig. 6:**
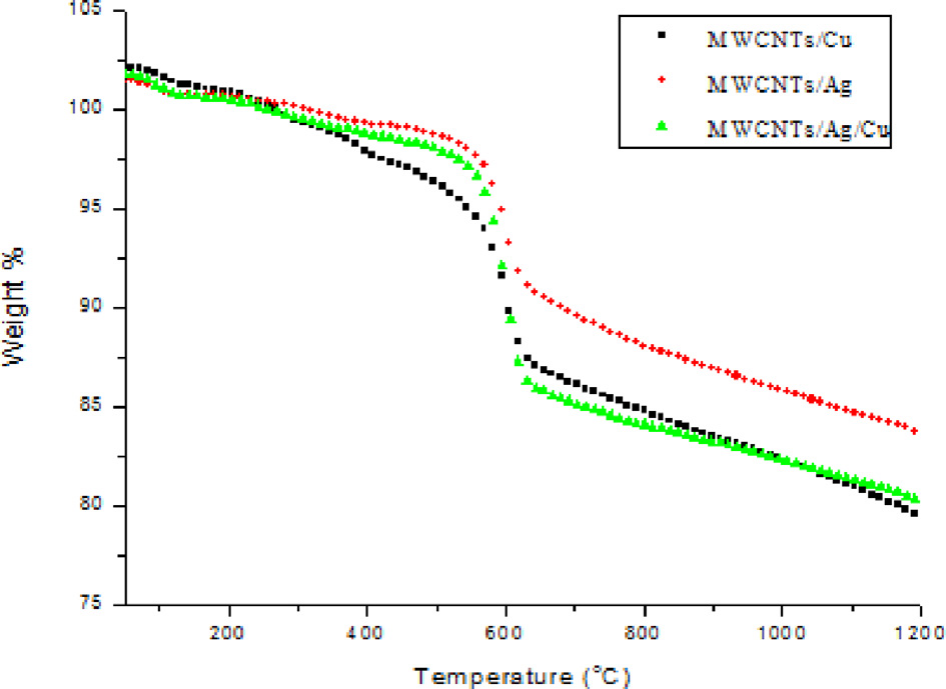
TGA of MWCNTs/NP hybrids.

The TGA curves of MWCNTs/Cu, MWCNTs/Ag and MWCNTs/Ag/Cu are shown in Fig. 6. This can be observed in all the samples that initial weight loss take place in range of 30–90 °C due to removal of water at ambient temperature. In MWCNTs/Cu, there is gradual weight loss within a temperature range of 90–579 °C which is due to physical damage consequential to the carbon content. Further drastic weight loss is observed in the temperature range of 579–620 °C. In the case of MWCNTs/Ag the similar type of behavior was observed with continuing weight loss in the range of 90–569 °C, followed by the sudden weight loss in the range of 569–626 °C. The MWCNTs/Ag/Cu showed almost the same trend as the other two samples with steady weight loss in the range of 90–563 °C and a sudden weight loss in the range of 563–623 °C. Maximum decomposition temperature is noted for MWCNTs/Cu i.e. 579 °C followed by MWCNTs/Ag and MWCNTs/Ag/Cu with maximum decomposition temperature of 569 °C and 563 °C respectively. The char yield in all the three samples is high due to high metal content. The char yield of 83.49wt% is observed for MWCNTs/Ag followed by MWCNTs/Ag/Cu and MWCNTs/Cu with char yields of 79.72wt% and 78.74wt% respectively.

It can be observed from Table 1 that MWCNTs/Ag showed decomposition at lower temperature compared to MWCNTs/Cu and maximum residual mass which represents the less thermal stability of the composite in air. The reason behind is behavior is the fact that Ag is an effective oxidation catalyst which can effectively split oxygen molecules and the resulting atoms are lightly adsorbed on the surface of MWCNTs (Alimohammadi et al., 2012). This also proves that the deposition of AgNp on MWCNTs accelerates the decomposition of MWCNTs more effectively as previously mentioned by Xim et al. (Neelgund and Oki, 2011).

**Table 1:** Degradation temperatures of synthesized hybrids.

Sample	To °C	T_10_ °C	T _50_ °C	Tmax°C	Char Yield (wt%)
CNTs/Cu	90	403	612	579	78.74
CNTs/Ag	90	533	630	569	83.49
CNTs/Ag/Cu	90	508	602	563	79.72
DGEBA/CNTs/Cu	-	336	387	328	22.29
DGEBA/CNTs/Ag	-	328	394	332	20.18
DGEBA/CNTs/Ag/Cu	-	336	387	328	20.88

**Fig. 7:**
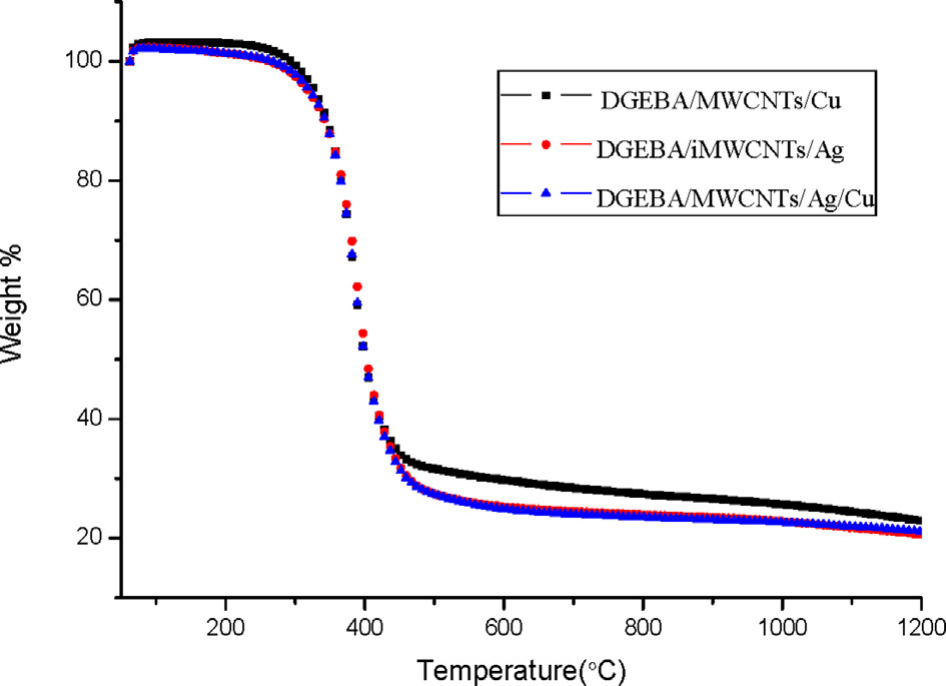
TGA of DGEBA/MWCNTs/NP hybrids.

The TGA graph of DGEBA/MWCNTs/Cu, DGEBA/MWCNTs/Ag and DGEBA/MWCNTs/Ag/Cu is shown in Fig. 7.

The presence of DGEBA causes the composites to show almost similar type of behavior with initial loss of water between 30-90 °C temperature ranges. The maximum decomposition temperature for DGEBA/MWCNTs/Cu, DGEBA/MWCNTs/Ag and DGEBA/MWCNTs/Ag/Cu are 328 °C, 332 °C and 330 °C respectively. There is gradual weight loss in all the three samples starting from 328-430 °C in DGEBA/MWCNTs/Cu, 332–426 °C in DGEBA/MWCNTs/Ag and 330–437 °C in DGEBA/MWCNTs/Ag/Cu. Maximum char yield is observed in DGEBA/MWCNTs/Cu of 22.29wt%.

The change in the thermal properties of the nano composites is due to incorporation of the CNTs along with the Cu and Ag nanoparticles. As it is a well-known fact that the thermal properties of CNTs is much higher due to its unique structure (Ruoff and Lorents, 1995). The incorporation of the Np on the surface also adds on the enhancement of the thermal properties due to the physical interaction between CNTs and the DGEBA.

**Fig. 8:**
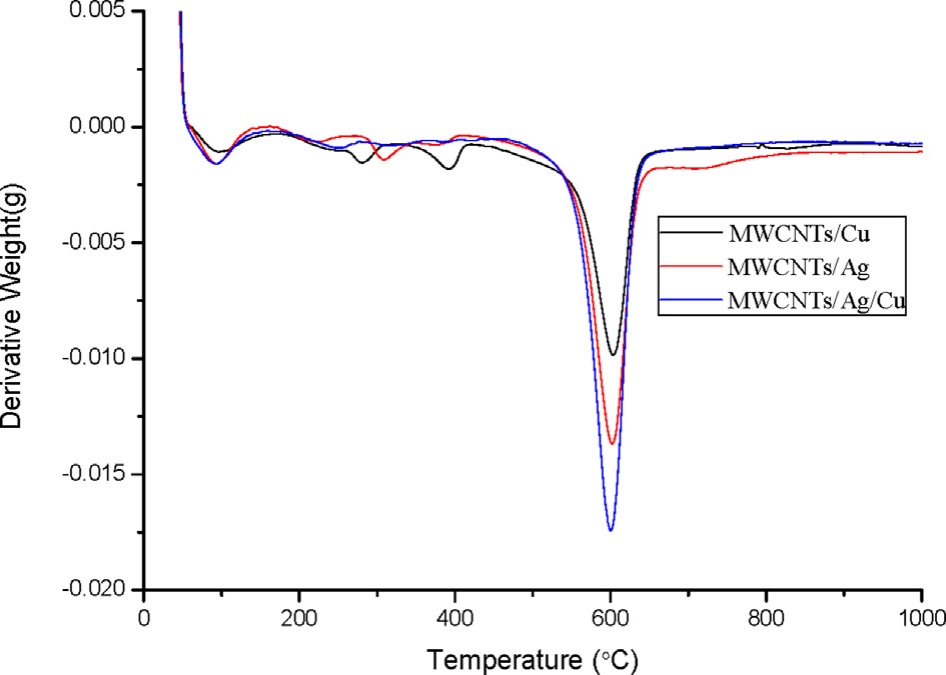
DTG of (a) MWCNTs/Ag(b) MWCNTs/Cu (c) MWCNTs/Ag/Cu.

The derivative thermogravimetric analysis (DTA) profiles represent the oxidation temperature as shown in Fig. 8.

The crystalline structure of MWCNTs composites shows a narrow oxidation peak within a temperature range of 560–610 °C. The oxidation peaks in the range of 200–300 °C corresponds to the oxidation of amorphous carbon (Vaisman et al., 2006). It showed that the MWCNT/Cu hybrids decompose at a much faster rate followed by MWCNT/Ag and MWCNT/Ag/Cu hybrids. It can be observed that the breadth of the derivative peak for MWCNT/Ag/Cu hybrids is slightly more in comparison to MWCNTs/Ag and MWCNTs/Cu due to the increased defects on graphitic network of the nanotube (Motchelaho et al., 2011).

### 3.5 XPS

**Fig. 9:**
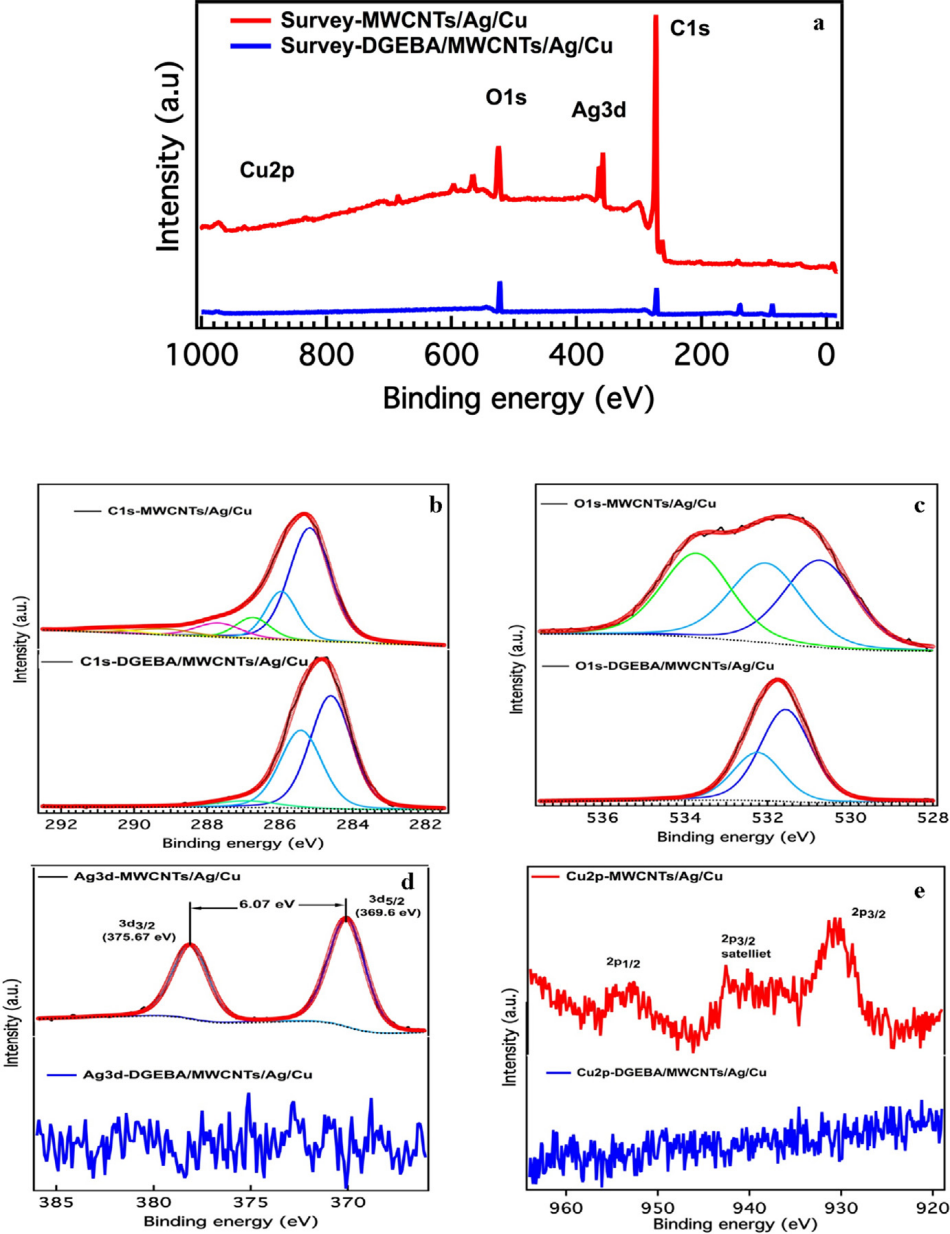
X-ray photoelectron spectroscopic images (a) survey scan of MWCNTs/Ag/Cu and DGEBA/MWCNTs/Ag/Cu (b) C1s (c) O1s (d) Ag3d (e).

The chemical composition and the presence of different functional groups in the MWCNTs/Np and DGEBA/MWCNTs/NP were also characterized by XPS as shown in Fig. 9. It was observed that the signals for C, O, Cu, Ag were clearly seen in the survey scan of MWCNTs/Ag/Cu, whereas no signal for Ag and Cu was observed in the DGEBA/MWCNTs/Ag/Cu (Fig. 9a). This observation further confirms that all the MWCNTs are fully covered in DGEBA and no molecule of Ag or Cu is present in the surface as XPS is the surface analysis technique. The C1s spectrum of MWCNTs/Ag/Cu is deconvoluted into six sub peaks (Fig. 9b). The first peak at 284.6 eV is assigned to aromatic carbon bonding (sp2) from hexagon walls of CNTs. The second peak located at 284.5 eV is related to C–CH sp^3^ bond indicates the defects in the aromatic structures. The other peaks positioned at 286.4 eV, 287.5 eV and 289.0 eV may be assigned to C–O, C=O and O–C=O respectively. The Plasmon π π^*^peak feature of CNTs has placed around 291.01 eV.

The C1s spectra obtained for DGEBA/MWCNTs/Ag/Cu was resolved into three peaks only, which shows the covering of DGEBA on the surface of MWCNTs/NP. The first peak at 284.5 eV is ascribed to C–C/C–H and peak centered at 285.5 eV is due to OH functionalities of DGEBA. The third peak appeared at 286.9 eV may be assigned to C>= functional groups. These assignments are also in good agreement with reported literature (Francisco et al., 2015).

The deconvoluted XPS O1s spectra of MWCNTs/Ag/Cu (Fig. 9c) show a peak at 533.8 eV is characterized by O–C, while the peak at higher bind energy at 532.3 eV describe the C=O/OH species. The third peak leveled 534.5 eV is indication of water/moisture content. The O1s spectra collected from as received DGEBA/MWCNTs/Ag/Cu is resolved into two peaks. The first peak at 531.6 eV is assigned to O=C/CH while the other peak at 532.2 eV is related to OH species, which again showed the anchoring of DGEBA on the surface MWCNTS/NP.

The presence of Ag3d peak in the wide-scan spectra of MWCNTs/Ag/Cu was clearly marked (Fig. 9d). The peaks having binding energy at 369.6 and 375.67 can be assigned to metallic Ag 3d_5/2_ and Ag 3d_3/2_ respectively. The Ag3d spectra also depicts the spin-orbit splitting, separated by 6.07eV, indicating the formation of metallic silver on the surface of MWCNTs. The boarding in peaks shows that Ag is slightly oxidized as reported in previous literature [40]. The XPS results of Cu2p as shown in Fig (9e), the main peaks are observed at 933.0 and 953.6 eV and are linked to the binding energy of Cu2p_3/2_ and Cu2p_1/2_ respectively. The appearance of these peaks confirms the formation of Cu^2+^ on the surface of the MWCNT/NP which is due to the surface oxidation of the hybrids. The strong satellite feature is also an indication of oxidation states of Cu (Cui and Wang, 2016).

## 4 Conclusions

MWCNTs/Ag/Cu and DGEBA/MWCNTs/Ag/Cu hybrids were synthesized successfully by chemical method. The result of this study designate that the attachment of NP on the surface of MWCNTs have improved the structural, morphological and thermal properties of MWCNTs whereas the chemical method used to incorporate the MWCNTs/NP in DGEBA was successful and physical bonding such as pi-pi interaction plays a trivial role in the incorporation of the hybrids. The FESEM images display that the epoxy has fully covered the MWCNTs, which is further confirmed by FTIR and XPS analysis. TGA shows that the MWCNTS/Cu hybrids decompose at a much faster rate whereas the Tmax of MWCNTs/Ag is higher. Change in the chemical nature of MWCNTs i.e. incorporation of NP along with their type was the influential factor determining the nanocomposite properties. It was analyzed that the dispersion of the MWCNTs in the matrix was the most vital step in the synthesis.

## Declarations

### Author contribution statement

Anila Iqbal, Jamaluddin Mahar: Performed the experiments.

Aamer Saeed: Conceived and designed the experiments; Wrote the paper.

Ayesha Kausar: Conceived and designed the experiments.

Muhammad Arshad: Analyzed and interpreted the data.

### Funding statement

This work was supported by HEC (Pakistan) under indigenous 5000 scholarship scheme-phase II.

### Competing interest statement

The authors declare no conflict of interest.

### Additional information

No additional information is available for this paper.
